# Association of Baseline Depressive Symptoms with Prevalent and Incident Pre-Hypertension and Hypertension in Postmenopausal Hispanic Women: Results from the Women’s Health Initiative

**DOI:** 10.1371/journal.pone.0152765

**Published:** 2016-04-28

**Authors:** Ruth E. Zambrana, Lenny López, Gniesha Y. Dinwiddie, Roberta M. Ray, Charles B. Eaton, Lawrence S. Phillips, Sylvia Wassertheil-Smoller

**Affiliations:** 1 Department of Women’s Studies, Consortium on Race, Gender and Ethnicity, University of Maryland, College Park, Maryland, United States of America; 2 Division of Hospital Medicine, University of California, San Francisco, California, United States of America; 3 African American Studies Department, University of Maryland, College Park, Maryland, United States of America; 4 Fred Hutchinson Cancer Research Center, Seattle, Washington, United States of America; 5 Family Medicine & Epidemiology, Alpert Medical School, Brown University, Providence, Rhode Island, United States of America; 6 Division of Endocrinology and Metabolism, Emory University School of Medicine, Atlanta, Georgia, United States of America; 7 Department of Epidemiology & Population Health, Albert Einstein College of Medicine, Bronx, New York, United States of America; Medical University Innsbruck, AUSTRIA

## Abstract

**Background:**

Depression and depressive symptoms are risk factors for hypertension (HTN) and cardiovascular disease (CVD). Hispanic women have higher rates of depressive symptoms compared to other racial/ethnic groups yet few studies have investigated its association with incident prehypertension and hypertension among postmenopausal Hispanic women. This study aims to assess if an association exists between baseline depression and incident hypertension at 3 years follow-up among postmenopausal Hispanic women.

**Methods:**

Prospective cohort study, Women’s Health Initiative (WHI), included 4,680 Hispanic women who participated in the observational and clinical trial studies at baseline and at third-year follow-up. Baseline current depressive symptoms and past depression history were measured as well as important correlates of depression—social support, optimism, life events and caregiving. Multinomial logistic regression was used to estimate prevalent and incident prehypertension and hypertension in relation to depressive symptoms.

**Results:**

Prevalence of current baseline depression ranged from 26% to 28% by hypertension category and education moderated these rates. In age-adjusted models, women with depression were more likely to be hypertensive (OR = 1.25; 95% CI 1.04–1.51), although results were attenuated when adjusting for covariates. Depression at baseline in normotensive Hispanic women was associated with incident hypertension at year 3 follow-up (OR = 1.74; 95% CI 1.10–2.74) after adjustment for insurance and behavioral factors. However, further adjustment for clinical covariates attenuated the association. Analyses of psychosocial variables correlated with depression but did not alter findings. Low rates of antidepressant medication usage were also reported.

**Conclusions:**

In the largest longitudinal study to date of older Hispanic women which included physiologic, behavioral and psychosocial moderators of depression, there was no association between baseline depressive symptoms and prevalent nor incident pre-hypertension and hypertension. We found low rates of antidepressant medication usage among Hispanic women suggesting a possible point for clinical intervention.

**Trial Registration:**

Clinicaltrials.gov NCT00000611

## Introduction

Investigators over the last two decades have produced an increasing body of knowledge on racial/ethnic hypertension disparities [1–4] and hypothesized that depression plays a mediating role in elevated blood pressure among adult women. [5–10] Depression and depressive symptoms are risk factors for hypertension (HTN) and cardiovascular disease (CVD). However the pathophysiologic mechanisms linking depressive symptoms to cardiovascular outcome has yet to be understood among Hispanic women. [11–14] Depression and anxiety are more common in women than men, increase with age, and menopause doubles the risk of hypertension even after adjusting for factors such as age and body mass index. [5,15] Several hypotheses have been proposed to explain this relationship: (1) Hypertension may be a mechanism through which depressive symptoms influence CVD pathogenesis; [7] (2) depression may contribute to hypertension, or conversely (3) women who are hypertensive may exhibit higher rates of depression. [15] Hispanic women (specifically of Puerto Rican and Mexican ancestry) across the life course have lower-income, less education, lower perceptions of social support that contribute to higher rates of depression and depressive symptoms compared to non-Hispanic White and other Hispanic subgroups. [16–19] A paucity of research exists that investigates the association between incident prehypertension and hypertension with depression among postmenopausal Hispanic women, and few include multiple psychosocial stressors, or use a longitudinal study design. [6]

Women’s response to life stressors play a role in the higher rates of depression. High rates of depression for Hispanic women include daily stressors such as living in low-resourced neighborhoods, and low social support with high levels of caregiving. [16,20,21] Hispanic women are more likely to experience stressful life events, less likely to receive mental health services, least likely to receive psychiatric medication during treatment, [16,22, 23] and are less likely to be aware of their HTN; receive treatment and or have their HTN controlled compared to other racial/ethnic women. [24] Comprehensive reviews of the role of psychosocial factors, namely low levels of social support, [25] stressful life events, low optimism, depression, and increased rates of caregiving responsibilities, [8,26–31] provide convincing evidence of their link with higher levels of systolic blood pressure. [6,14] Although social support has been shown to reduce stress, improve adherence to medication, and buffer disease severity and depression, [32,33] increased care giving responsibilities of family members and a pessimistic attitude play a significant role in predicting hypertensive risk. [34,35] These psychosocial predictors of depression have not been included in most prior epidemiologic studies. The contribution of this study is its ability to assess known psychosocial correlates of depression that have a significant association with depression and hypertension. We seek to assess if an association exists between baseline depression and incident hypertension at 3 years follow-up among postmenopausal Hispanic women.

## Methods

The Women’s Health Initiative (WHI) is a large, multiethnic, 40-center study funded by the National Heart, Lung, and Blood Institute (NHLBI)that focuses on strategies for preventing heart disease, breast and colorectal cancer, and osteoporotic fractures in postmenopausal women. The total sample includes 161,808 women aged 50–79 years. Self-reported race/ethnicity includes 82.5% non-Hispanic White; 9.0% Black or African American; 4.0% Hispanic, predominantly Mexican American; 2.6% Asian or Pacific Islander; and 1.5% other. For the analyses, we include 4,680 Hispanic women who participated in the WHI observational and clinical trial studies at baseline (1994–1998) and at third-year follow-up and for whom blood pressure was measured at baseline and year 3. Data were collected during a baseline screening visit and during a third year visit; the data included physical measurements and questionnaires related to medical history, family history, and behavioral factors. A full description of the WHI study is presented elsewhere. [36,37] The dependent variables were prehypertension and hypertension analyzed in separate models. Blood pressure was measured at the first screening clinic visit by certified staff with the use of standardized procedures and instruments; it was measured in the right arm with a mercury sphygmomanometer after the participant was seated and had rested for 5 minutes. [36] The cuff, was inflated to 30 mm Hg above palpated systolic blood pressure (SBP). SBP was defined as the pressure level at which the first of two Korotkoff sounds occurred in appropriate rhythm and diastolic blood pressure (DBP) was the phase V Korotkoff value. The average of 2 blood pressure readings obtained at least 30 seconds apart, was used for analysis. Hypertension was defined in women who reported that they were told by a doctor they had high blood pressure, and/or those who were prescribed medications for hypertension, and/or those whose systolic blood pressure was ≥140 mm Hg, and/or those whose diastolic blood pressure was ≥90 mm Hg. [1] Participants with prehypertension were those women whose systolic/diastolic blood pressure was 120-139/80-89 mm Hg with no self-report of medication prescribed for hypertension as defined by the Seventh Report of the Joint National Committee on Prevention, Detection, Evaluation, and Treatment of High Blood Pressure (JNC7). [38] Participants with normal blood pressure had systolic/diastolic blood pressure < 120/80 mm Hg and no self-report of medication for hypertension.

At baseline, participants completed a psychosocial questionnaire using 6-items from the Center for Epidemiological Studies Depression Scale (CES-D) scale to assess depressive symptoms in the past week and two items from the Diagnostic Interview Schedule (DIS) regarding a history of depressive symptoms in the past year. [19,39,40] Women were asked in the CES-D to report how often, in the past week, they had felt depressed; had restless sleep; enjoyed life; had crying spells; felt sad; and felt that people disliked them. Items were scored as: rarely or none of the time (<1 day); some or a little of the time (1–2 days); occasionally or a moderate amount of time (3–4 days); and most or all of the time (5–7 days). The 2 items derived from the DIS asked: “‘In the past year, have you had 2 weeks or more during which you felt sad, blue, or depressed, or lost pleasure in things that you usually cared about or enjoyed?”; ‘‘Have you had 2 years or more in your life when you felt depressed or sad most days, even if you felt okay sometimes?”; and ‘‘If yes, have you felt depressed or sad much of the time in the past year?”

Based on prior research, [9,19,41] we constructed three measures of depression. The CES-D items measured current depressive symptoms at baseline and were scored from 0 to 3, with a score of 0 indicating the participant never or rarely experienced the depressive symptoms and a score of 3 indicating the symptom was experienced most or all of the time. A summary measure of current depressive symptoms at baseline summed the responses to the CES-D and categorized a score of 5 or higher (of a possible total 18) as indicative of symptoms of depression. In a second measure, we combined the DIS items, so that a woman who responded ‘yes’ to both questions was considered to have a prior history of depression. We constructed a third measure that included items from the CES-D and DIS and a logistic regression algorithm [39] was used to compute the Burnam score indicating probability of depression. The Burnam score ranges from 0 to 0.99 with a score higher than 0.06 defining high depressive symptomatology. [19] This threshold score is not itself a measure of clinically diagnosed depression but is well correlated with clinical depression. [41] We coded antidepressant use at baseline (yes/no) from a detailed medication inventory at that visit.

Our assessment of psychosocial factors included four validated scales that measured stressful life events, social support, optimism, and caregiving responsibilities. An index of stressful life events was created with 11 items, derived from the Alameda County Epidemiologic Study, [42] and modified for the Beta Blocker Heart Attack Trial. [43] A summary score (range: 0–33) was computed as the count of the number of events weighted by the participant’s appraisal of stress attributed to each event with higher scores indicating a greater number of stressful events. The stress-weighted number of life events was divided into four categories of events (0, 1–2, 3–4, 5–33). Social support was assessed using 9 items from the Medical Outcomes Study questionnaire. [44] Participants ranked on a 5-point scale how often emotional, affection, and tangible and positive interaction support domains were available. The total summary score ranged from 9 to 45, where a higher score indicated more social support. For analysis, we recoded the scores as lower (9 to 33), medium (34 to 40), and higher levels of support (41 to 45). Optimism was measured using the Life Orientation Test-Revised (LOT-R) six item scale. [45] Responses were coded on a 5-point scale, and categorized as low (6–23), medium (24, 25), and high (26–30). Caregiving responsibility was based on a two-part item derived from the Cardiovascular Health Survey (none/less than once a week, 1–2 times per week, ≥3 times per week). [46]

All participants provided written informed consent. Institutional review board approval was obtained from each of the participating study centers and from the Fred Hutchinson Cancer Research Center, which currently serves as the IRB of record for the WHI.

### Analyses

All statistical analyses were performed using SAS System for Windows version 9.3 (SAS Institute, Cary, NC). Descriptive analyses evaluated the unadjusted baseline associations of hypertension status (normotensive, prehypertensive, or hypertensive) by demographic, clinical, behavioral, and psychosocial characteristics of the participants, and the three depression measures. For our main analysis, we dichotomized continuous Burnam scores at the standard threshold of 0.06 to identify women experiencing symptoms consistent with depressive disorders or probability of depression, including major depression and dysthymia [1,19,47]

We used multinomial logistic regression models to evaluate: hypertension status at baseline in relation to each of three measures of depression, adjusted for the effects of other explanatory variables and potential confounders included in each model; and the association of incident prehypertension and hypertension at year 3 with the probability of depression, using baseline Burnam score in multivariable models. Associations were presented as odds ratios (OR) and 95% confidence intervals (95% CI).

A set of baseline covariates was selected a priori to adjust for potential confounding. These included age at enrollment (50–64, 65–79); level of education (less than high school, high school degree/GED/vocational or training school, some college, and college graduate or above); personal medical history of cholesterol levels requiring pills, treated diabetes, and history of CVD; BMI (<25 kg/m^2^, 25 to <30 kg/m^2^, ≥30 kg/m^2^); family history of adult diabetes, stroke or myocardial infarction (computed as the sum of conditions reported, scores ranging from 0 to 3); health insurance coverage (yes/no); smoking status (never smoked/past smoker, current smoker; physical activity assessed in metabolic equivalent of task (MET)-hours per week (0 to <3.0, 3.0 to <11.75, ≥11.75); alcohol intake (nondrinker/past drinker, < 7 drinks per week, ≥7 drinks per week); and antidepressant use (yes/no). In addition, we separately evaluated the effects of four self-reported psychosocial measurements: stressful live events, social support, optimism and caregiving responsibilities.

## Results

The mean age at baseline of participants by hypertension status was: normotensive: 58.3 years (SD 6.17), prehypertensive: 60.2 years (SD 6.7) and hypertensive: 62.3 years (SD 6.8). Prevalence of prehypertension and hypertension at baseline was lower in participants with some college education or higher compared to those with a lower education attainment (Table 1).

**Table 1:** Baseline Characteristics of Hispanic Women in the WHI Cohorts, by Baseline Hypertension Status.

		Normotensive (N = 1564)		Prehypertensive (N = 1581)		Hypertensive (N = 1535)	
		No.	%	No.	%	No.	%
Education	Less than high school	306	20.0	393	25.3	434	28.7
	High school diploma or GED, vocational or training school	437	28.5	450	28.9	444	29.4
	Some college or associate degree	400	26.1	394	25.3	344	22.8
	College graduate and above	390	25.4	319	20.5	289	19.1
Age at WHI enrollment	50–64	1293	82.7	1161	73.4	942	61.4
	65–79	271	17.3	420	26.6	593	38.6
Body-mass index (kg/m2), baseline	<25	551	35.5	348	22.2	273	18.0
	25 - <30	632	40.7	631	40.2	549	36.3
	> = 30	368	23.7	592	37.7	691	45.7
Family history of diabetes, stroke, MI (number of conditions)	0	474	30.7	458	29.3	354	23.6
	1	552	35.8	570	36.4	538	35.8
	2	375	24.3	390	24.9	449	29.9
	3	141	9.1	146	9.3	162	10.8
History of high cholesterol requiring pills	No	1282	89.5	1262	86.4	1150	79.9
	Yes	150	10.5	198	13.6	290	20.1
Treated diabetes (pills or shots)	No	1503	96.2	1501	94.9	1378	89.9
	Yes	59	3.8	80	5.1	155	10.1
History of cardiovascular disease	No	1338	91.5	1340	90.1	1181	81.1
	Yes	125	8.5	147	9.9	276	18.9
Insurance Status	Public only (Medicare/Medicaid)	112	7.5	169	11.2	217	14.9
	Private only	871	58.6	777	51.7	677	46.6
	Public/Private combination	107	7.2	156	10.4	230	15.8
	Military/VA sponsored and/or other	127	8.5	106	7.0	112	7.7
	No insurance	269	18.1	296	19.7	216	14.9
Smoking status	Never smoked, past smoker	1408	91.5	1467	93.9	1429	94.3
	Current smoker	131	8.5	96	6.1	87	5.7
Moderate-strenuous physical activity > = 20 min	No activity/some activity	920	61.8	995	66.2	1022	69.3
	2 or more episodes per week	568	38.2	509	33.8	452	30.7
Total energy expenditure/week, MET-hours	0-<3.0	493	33.1	548	36.4	545	37.0
	3.0-<11.75	468	31.5	455	30.3	507	34.4
	> = 11.75	527	35.4	501	33.3	422	28.6
Alcohol intake	Non-drinker	240	15.6	297	19.1	333	22.1
	Past drinker	329	21.4	351	22.5	379	25.1
	<7 drinks per week	893	58.0	826	53.0	735	48.7
	7+ drinks per week	78	5.1	84	5.4	63	4.2
History of depression	No	1221	82.0	1201	79.4	1164	79.6
	Yes	268	18.0	312	20.6	298	20.4
Currently depressed at baseline	No	1077	73.7	1076	72.4	1024	71.6
	Yes	384	26.3	410	27.6	406	28.4
Taking antidepressants at baseline	No	1469	93.9	1497	94.7	1423	92.7
	Yes	95	6.1	84	5.3	112	7.3
Depression (shortened CES-D/DIS score > = 0.06)	No	1166	80.8	1173	80.8	1098	78.3
	Yes	277	19.2	278	19.2	305	21.7
Caregiving frequency, baseline	None, < 1 time per week	1004	65.4	1010	65.0	971	64.7
	1–2 times per week	235	15.3	222	14.3	236	15.7
	3+ times per week	297	19.3	323	20.8	293	19.5
Life event construct (0–3 scoring, accounting for intensity of experience), baseline	None	269	18.2	252	17.0	251	17.8
	1–2	287	19.5	300	20.2	287	20.3
	3–4	299	20.3	334	22.5	348	24.6
	5+	619	42.0	599	40.3	528	37.3
Social Support Construct, baseline	9–33 (low)	609	40.9	607	40.3	618	43.1
	34–40	458	30.8	437	29.0	432	30.1
	41+ (high)	421	28.3	461	30.6	385	26.8
Optimism construct, baseline	<24 (low)	919	62.6	942	63.5	951	66.9
	24–25	253	17.2	274	18.5	232	16.3
	> = 26 (high)	296	20.2	268	18.1	238	16.7

For each characteristic, the numbers of women may sum to less than the total due to missing data.

Women enrolled in WHI at an older age, those with insurance, a BMI ≥ 30, taking high cholesterol and diabetes medications, a family and/or personal history of cardiovascular disease were more likely to have prehypertension or hypertension at baseline. Most participants were never or past smokers and were slightly more likely to have prehypertension or hypertension. However, women who had <7 drinks per week were less likely to have prehypertension or hypertension. Participants with ≥ 2 episodes of moderate-strenuous physical activity ≥ 20 minutes or ≥ 11.75 MET/hr/week were less likely to have prehypertension or hypertension. Of 4,680 Hispanic women, 26% met current depression criteria and 19% had a history of depression at baseline. About 12% reported both current depression and history of depression. There were no differences in baseline history of depression, current depressive status, or use of antidepressants at baseline across hypertension categories. Finally, there were no differences across measures of stressful life events, social support, optimism and caregiving by hypertension status.

After adjustment for the full set of demographic and clinical variables, we observed a modest association between a history of depression and prehypertension at baseline (OR 1.27, 95% CI: 1.01, 1.61). Age-adjusted multinomial models found an association between baseline depression (measured by shortened CES-D/DIS (score > = 0.06) and baseline hypertension (OR 1.25, 95% CI: 1.04, 1.51) (Table 2).

**Table 2:** Associations of Depression with Baseline Prehypertension and Hypertension.

**Prehypertension**					
	Model 1	Model 2	Model 3	Model 4	Model 5
Variable	**OR (95% CI)**	**OR (95% CI)**	**OR (95% CI)**	**OR (95% CI)**	**OR (95% CI)**
Depression (shortened CES-D/DIS score > = 0.06)	1.03 (0.85, 1.24)	1.01 (0.84, 1.22)	1.02 (0.84, 1.24)	1.00 (0.81, 1.23)	1.01 (0.79, 1.29)
Currently depressed at baseline	1.09 (0.93, 1.29)	1.04 (0.88, 1.24)	1.05 (0.89, 1.25)	1.00 (0.83, 1.21)	1.02 (0.82, 1.26)
History of depression	1.21 (1.01, 1.46)	1.18 (0.98, 1.41)	1.19 (0.99, 1.44)	1.20 (0.98, 1.48)	1.27 (1.01, 1.61)
**Hypertension**					
	Model 1	Model 2	Model 3	Model 4	Model 5
Variable	**OR (95% CI)**	**OR (95% CI)**	**OR (95% CI)**	**OR (95% CI)**	**OR (95% CI)**
Depression (shortened CES-D/DIS score > = 0.06)	1.25 (1.04, 1.51)	1.19 (0.99, 1.44)	1.17 (0.96, 1.42)	1.08 (0.87, 1.34)	1.07 (0.83, 1.38)
Currently depressed at baseline	1.17 (0.99, 1.39)	1.08 (0.91, 1.28)	1.06 (0.89, 1.26)	0.92 (0.76, 1.12)	0.90 (0.72, 1.14)
History of depression	1.23 (1.02, 1.49)	1.16 (0.96, 1.40)	1.13 (0.93, 1.38)	1.05 (0.84, 1.31)	1.08 (0.84, 1.39)

Model 1: Age Adjusted

Model 2: Age + Education Level

Model 3: Model 2 + Antidepressant Use

Model 4: Model 3 + Behavior variables: (smoking status, total energy expenditure/week, alcohol intake) + insurance + Clinical variables: (BMI; family history of diabetes, stroke, or MI; high cholesterol requiring pills; treated diabetes; history of CVD)

Model 5: Model 4 + caregiving, stressful life events, social support and optimism

A similar association between history of depression and baseline hypertension was observed (OR 1.23, 95% CI: 1.02, 1.49). These findings were attenuated to the null in fully adjusted models. No associations were found between baseline hypertension status and measures of stressful life events, social support, optimism and caregiving (not shown), nor with baseline depression and incident prehypertension at year 3. Incident hypertension at year 3 was associated with baseline depression after adjustment for demographic characteristics (age, education), health care insurance, and behavioral factors (OR 1.74, 95% CI: 1.10, 2.74), but the association was attenuated after further adjustment for clinical factors such as body mass index (BMI) (OR = 1.53; 95% CI 0.95–2.46) (Table 3).

**Table 3:** Association of Baseline Depression ^a^ and Incident Prehypertension and Hypertension at Year 3 in Women who were Normotensive at Baseline.

Model	Prehypertension	Hypertension
Age-adjusted	0.84 (0.61, 1.15)	1.62 (1.06, 2.47)
Age and education adjusted	0.83 (0.60, 1.14)	1.55 (1.01, 2.38)
Age, education, and clinical variables ^b^	0.80 (0.58, 1.12)	1.38 (0.88, 2.17)
Age, education, insurance, and behavior variables ^c^	0.85 (0.60, 1.19)	1.74 (1.10, 2.74)
Age, education, insurance, and behavior + clinical variables	0.83 (0.58, 1.18)	1.53 (0.95, 2.46)

^a^ Depression was measured by the shortened CES-D/DIS (score > = 0.06) at baseline. 1248 women had complete covariate information for the full model.

^b^ Clinical variables include BMI; family history of diabetes, stroke, or MI; high cholesterol requiring pills; treated diabetes; history of CVD.

^c^ Behavior variables include smoking status, total energy expenditure/week, alcohol intake.

While the psychosocial variables of social support, optimism and stressful life events were not found to be associated with baseline hypertension, they were associated with depression and therefore could be effect modifiers on the relationship of depression with prehypertension and hypertension (p < .001). We therefore stratified our results by social support, optimism, and stress life events and evaluated the association of depression on incident prehypertension and hypertension. Stratified analyses by social support, optimism, and stress life events and the association of depression on incident prehypertension and hypertension demonstrated no effect modification by these factors.

## Discussion

Our analyses reveal that Hispanic women have high levels of baseline depression relative to other Hispanic cohorts, [48] and education moderates these depression rates. In this study, baseline depression and history of depression were not associated with incident prehypertension or hypertension. However, psychosocial variables (life events, social support and optimism) were associated with baseline depression in unadjusted analyses. This is the first study to include additional psychosocial variables in analyses of hypertension risk and these psychosocial variables have been observed to be associated with depression among urban, racial/ethnic and poor women, [49] and with cardiovascular events. [7,50] Among Hispanics, high rates of depression compared to other racial/ethnic groups nationally are associated with history of CVD, and increasing number of CVD risk factors that strongly correlate with psychosocial stressors and socioeconomic status. [10,17]

Hispanic women across the life course have consistently demonstrated high rates of depression (ranges from 22.3% to 43.1%) that are associated with increased hypertension risk.[8,10,17,51] We observed only modest associations of depression at baseline and incident hypertension in 3-year follow-up. Our findings are consistent with prior longitudinal studies [52] which have found mixed associations between baseline depressive symptoms and incident hypertension. The Whitehall II cohort [53] and CARDIA demonstrated a positive association [54] while the Multi-Ethnic Study of Atherosclerosis (MESA) cohort found small associations between baseline depressive symptoms and increases in blood pressure but no statistically significant association with incident hypertension. [51] Although our study is unique by focusing only on Hispanics, it is unknown if the differences in our findings could be attributable to differences in age distributions in other population cohorts, follow-up time, depression measure used, or possibly socio-ethnic differences in the experience and reporting of depression.

The psychosocial variables considered shed some light on the complex yet indirect moderating role that these factors may exert on hypertension risk and confirm the findings of other studies. One study of 2,564 Mexican Americans aged 65 or older showed an association between high positive emotion and lower blood pressure among older Mexican Americans. [55] In contrast, pessimism heightens risk for self-reported, treated and incident hypertension, [56] and significant interactions are reported between pessimism, socioeconomic status (SES) and hypertension. [30,57] In our study, the majority of respondents who reported low rates of social support (61–63%) also had the highest rates of current depression and history of depression at baseline suggesting that social support may not be able to “buffer” stress from adverse life events. [58]

A recent study of 15,864 Hispanics confirms the association of depression with education level and Hispanic subgroup with higher rates observed for those with less than a high school education among those aged 45–64 similar to our study. [17] Another study of Hispanic women (<high school) found that 11% reported at least one major depressive disorder in the past 12 months while 12% rated their mental health status as fair or poor. [22] In our study about one-third of women with less than a high school education reported a history of (24.5%) and current depression (35.3%) compared to respondents who are college graduates or above (19.8% and 14.5% respectively). Unadjusted analyses confirm that education was inversely associated with depression with a significant decrease in rates of depression as education increases. These high rates of depression may be due to differences in awareness and reporting of depressive symptoms that are influenced by higher educational attainment and income, and higher rates of health care insurance coverage that may increase use of mental health services and psychotropic medications. [59] Alternatively Hispanic women’s higher prevalence of depression may reflect more exposure to stressful workplace events, discrimination [60,61] and living in unsafe neighborhood contexts. [62] In our study, higher levels of current depression at baseline were associated with lower levels of antidepressant use (7%) and lack of health insurance (15%). Moreover, Hispanic patients may have their medications less intensified than other racial/ethnic groups.[63]

Our study has several limitations. The Hispanic sample is predominantly of Mexican-origin and these data may not be representative of other Hispanic subgroups. All women were postmenopausal and our findings cannot be generalized to younger reproductive age women. This cohort were healthier at baseline than women of this age in the U.S. population, as indicated by lower baseline prevalence of diabetes, hypertension, CVD, high cholesterol levels that required medication, and cigarette use. [64] Education levels were higher among WHI participants and fewer women reported no leisure-time physical activity compared to other cohorts with Hispanics. These demographic and health findings may be attributed to the voluntary recruitment strategy that was used in the WHI studies. Although we accounted for traditional CVD risk factors in multivariable regression models, we were unable to include all possible confounders thus preventing any causal conclusions about incident hypertension. Additionally, we did not include use of medication in management of prehypertension or initial drug therapy with compelling indications as suggested by the JNC7 report. Notwithstanding these limitations, our longitudinal study is the largest study to date of older Hispanic women which included physiologic, behavioral and psychosocial moderators of depression.

Future studies need to be mindful of evaluating psychosocial factors such as social support to assess its moderating effect by SES and to parse out SES by nativity and ethnic background/ancestry to disentangle effects of depression on hypertension, [4,65] as Hispanic women report less control over their lives and experience more negative life events than men and majority groups. [66] Yet, questions remain regarding what are the direct and indirect effects of psychosocial variables on hypertension development, management and control for Hispanic populations. [6,14] Primary prevention is an important goal in the detection and management of HTN among Hispanic populations and differences by nativity, geographic region and Hispanic subgroup are significant moderators of hypertension. [3,17] We found low rates of antidepressant medication usage that confirms other studies suggesting an additional clinical intervention to improve the higher rates of depressive symptoms among Hispanic women. [17] Further analyses that included psychosocial variables correlated with depression did not alter findings suggesting the complex role these factors may exert on cardiovascular health. Elucidating the complex pathways of depression’s effect on hypertension may lead to appropriately tailored interventions among Hispanic women.
